# A history-based method to estimate animal preference

**DOI:** 10.1038/srep28328

**Published:** 2016-06-28

**Authors:** Caroline Marques Maia, Gilson Luiz Volpato

**Affiliations:** 1 Laboratory of Animal Physiology and Behaviour, Department of Physiology, Caunesp, Institute of Biosciences (IB), Sao Paulo State University (UNESP), Botucatu, SP, Brazil

## Abstract

Giving animals their preferred items (e.g., environmental enrichment) has been suggested as a method to improve animal welfare, thus raising the question of how to determine what animals want. Most studies have employed choice tests for detecting animal preferences. However, whether choice tests represent animal preferences remains a matter of controversy. Here, we present a history-based method to analyse data from individual choice tests to discriminate between preferred and non-preferred items. This method differentially weighs choices from older and recent tests performed over time. Accordingly, we provide both a preference index that identifies preferred items contrasted with non-preferred items in successive multiple-choice tests and methods to detect the strength of animal preferences for each item. We achieved this goal by investigating colour choices in the Nile tilapia fish species.

A crucial challenge in the field of animal care is how to identify animal welfare along a continuum from poor to good^1^,^2^. Although identification of health or disease states is undoubtedly necessary for this purpose, this issue requires deeper analyses. A healthy state does not assure good welfare, as even a stressed animal might be in good health^3^,^4^. This problem has spurred philosophical controversies over whether animals are aware of suffering or discomfort^5^,^6^,^7^,^8^,^9^. In this debate, Marian Dawkins^8^,^10^ has proposed that we should provide the animals what they want. This obvious and parsimonious proposal compels us to understand what animals prefer, which is not an easy task^11^.

Most studies have evaluated animal preferences based on a few choice tests (*e.g*.^12^,^13^,^14^,^15^,^16^,^17^), thus providing similar considerations of choices and preferences. Moreover, most researchers have used the frequency/time spent in the available options of just one trial or summed/averaged data from few trials to determine what animals prefer. However, animal choices may differ in subsequent tests^18^,^19^. Thus, in such few days of testing, a spurious choice response from one specific test could significantly impact the overall preference response when equally summing or averaging data over tests. This implies momentary choices may bias the overall preference response of the animal. Furthermore, most studies have evaluated the preferences at a group level, regardless of the individual variations in such responses (*e.g*.^20^,^21^,^22^,^23^,^24^,^25^). However, various studies have demonstrated significant individual variability even when preference responses are analysed from a few tests (*e.g*.^26^,^27^,^28^,^29^).

In this context, we propose a history-based method to determine individual preferences from multiple-choice tests. Because internal motivation and development might change over time, we assumed that in the context of animal welfare, animals have variable wants in addition to their innate needs. Thus, detecting preferences to give to these animals a better environment should assume that animal preferences might change over tests. For these reasons, animal preferences are best determined by a cumulative set of tests. Thus, our history-based method focuses on a consecutive set of tests that incorporates the more recent decisions of the animals. This method was decided after considering that the more recent the choice is made, the more weight it has on the preference response. This method was conceptually developed and applied over a 10-day period of successive daily tests. We used the fish Nile tilapia, *Oreochromis niloticus* (L.), in background colour choice tests. This species has been described to prefer yellow light (2-day test^30^), and some colours have been demonstrated to affect its behaviour^31^,^32^,^33^,^34^ and physiology^32^,^34^,^35^.

## Materials and Methods

The procedures of this study were in accordance with the guidelines stated by the Ethical Principles in Animal Research adopted by the Brazilian College of Animal Experimentation (COBEA) and were approved by the Bioscience Institute/UNESP Ethical Committee for Animal Research (CEEA), protocol # 220. Our research procedures were carried out strictly in accordance with these approved guidelines.

### Animals and Holding Conditions

We used juvenile Nile tilapia, *Oreochromis niloticus* (Linnaeus, 1759), with no sex determination, provided by commercial hatcheries. The fish were maintained in indoor tanks (2 fish/10 L in 1000–1700 L tanks) for at least 30 days prior to experimentation. In this period, we fed the fish commercial tropical fish pellets once per day (5% of biomass/day). The water temperature was approximately 26 °C; pH ≅ 6.8; ammonium <0.3 ppm; and nitrite <0.05 ppm. The aeration was constant, and the photophase (white light) was set from 07:00 h to 19:00 h.

### Theoretical Assumptions

1. Because animals might vary their preferences over time^18^,^19^, we considered each animal’s history of successive choice tests instead of only the last few tests.
2. Due to practical purposes and because an animal’s preference might change over time, we required the most recent data related to an animal’s preferences to provide better environments and conditions. The most recent choices should have a higher impact on the calculations of the animal’s preferences. Focusing on current preference of an animal, successive choice tests provide results of a time sequence of momentary choices. However, the main focus is to know what is the preference of an animal in a specific moment, but considering its whole history. A choice test run at that moment is based on a very specific condition of the animal, which is more susceptible to be influenced by several momentary factors. If this result is considered to provide the animals with a better environment, then a response at a time would be the base for longer decisions about animal welfare conditions. To counteract this, we added to the result at a time (present time) other animal choices from previous tests. This balances our decision for more reasonable information about animal’s wants. The results of recent tests overestimate momentary factors influencing choices and results of older tests overestimate the previous choices of that animal. At each new choice test, older tests become more frequent, so that overweighing the recent tests could better balance this profile.
3. The effect of any spurious high choice in one or a few tests should be minimized for calculations of an animal’s preferences to avoid bias.
4. An animal might prefer more than one choice item. The lower the number of preferred items, the stronger the preference should be. Thus, different calculations for animals with only one preference item and more than one preferred item should be considered.
5. The technical approach to obtain data should be feasible in practical terms for zookeepers dealing with captive animals. Thus, we employed an easy procedure for registration of animal choices: the number of visitations in an instantaneous sampling scheme during a fixed observation interval^36^.
6. We also considered the need to know which item(s) an animal preferred and which animal had stronger preferences when compared with conspecifics. Moreover, the method to detect an animal’s preference should consider individual variations among the preferred items, once various studies have demonstrated significant individual variability^26^,^27^,^28^,^29^.

### General Design

Each fish was carefully caught in the stock tanks and individually isolated in glass aquaria (40 × 20 × 25 cm) and monitored for 24 h before the choice tests began. Only fish that appeared to be unstressed after isolation (paler eyes^37^) were used. The fish sample consisted of 24 juveniles (mean total length = 6.91 cm ± 0.41 cm; mean body weight = 5.51 g ± 0.96 g), with no sex determination. After the fish were transferred to a test apparatus, we monitored each fish’s choices for four compartments of different background colour (yellow, green, red or blue). Over 10 consecutive days, we measured animal choices every 30 s for 1 h each day and recorded the frequencies that the fish visited each compartment (instantaneous sampling^36^). In each test day, fish were video recorded between ~08:30 h to 09:30 h or ~09:45 h to 10:45 h. In the first period, we filmed 4 fish simultaneously from above, each fish placed in individual test apparatus. In the next period, we filmed more 4 fish in the same conditions. Thus, we repeated all these procedures more two times to reach 24 tested fish. The position of the test apparatus in the lab was constant during each successive test for each fish tested. From these data, we developed a method to separate preferred backgrounds from non-preferred backgrounds and calculate a preference index (PI) and a preference rate (PR), thus separating individuals based on the intensities of their preference responses.

### Apparatus and Procedures

**Figure 1:**
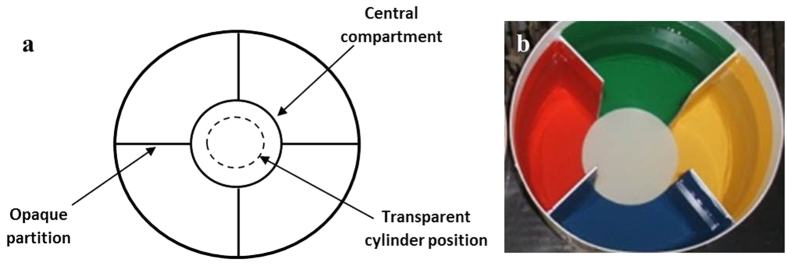
Upper view of test apparatus. (**a**) Scheme indicating four compartments with the same volume and area. Aquarium: 40-cm diameter; ~15-cm height of water column; opaque internal and external walls. (**b**)Photo depicting background colours offered as choice items. Compartment colours were obtained by addition of coloured adhesive plastic for the fourth peripheral compartments and by a circular white perplex in the central compartment.

The fish were introduced into the central compartment in a transparent 8-cm-diameter cylinder of the respective test apparatus, a cylindrical aquarium of 40-cm diameter with a water column of 15-cm height (~6.3 L; see Fig. 1). After a 5-min acclimation period, we gently removed the cylinder and video-recorded the fish from above for 1 h each day. Despite this 5 min of space restriction, the fish was able to freely move and, in a previous study^38^, 5 min of a stronger confinement was not stressful for this species. Every 30 s the fish position was recorded during the 1 h period resulting in 120 measurements per daily test. The fish was considered to be in a compartment when its eyes were inside the compartment. When the eyes were between two compartments, the chosen compartment was the one containing the majority of the fish body. After each test, we returned the fish to the respective isolation aquarium until testing the following day. We repeated these procedures each morning over 10 consecutive days.

In the isolation holding aquaria, aeration was constant, and 30% water changes were performed 1 to 3 times per week by siphoning water out and replacing this volume when the fish were in the test apparatus to maintain good water quality. The photophase was maintained from 08:00 h to 18:00 h and consisted of white light. For the test apparatus, aeration ceased during video recordings; the intensity of the light (white) at the water surface was ~150 Lux, and the water temperature was 25.8 ± 0.87 °C. Feeding was provided once a day in the isolation aquarium after each choice test.

### Calculations

**Figure 2:**
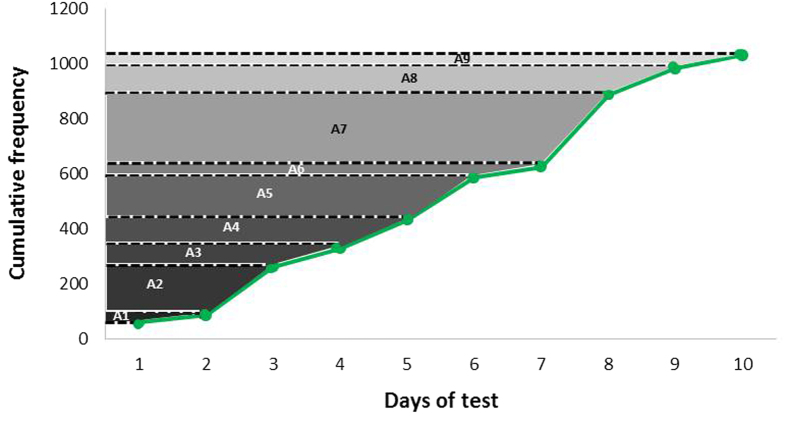
Schematic view of area calculations. See impacts on the most recent choices in a series of consecutive choice tests. Data obtained from fish 6 to visualize calculations of the areas (A1 to A9) above the line of cumulative frequencies. This assumption implies that unit 1 will always result in a higher area in subsequent tests.

**Figure 3:**
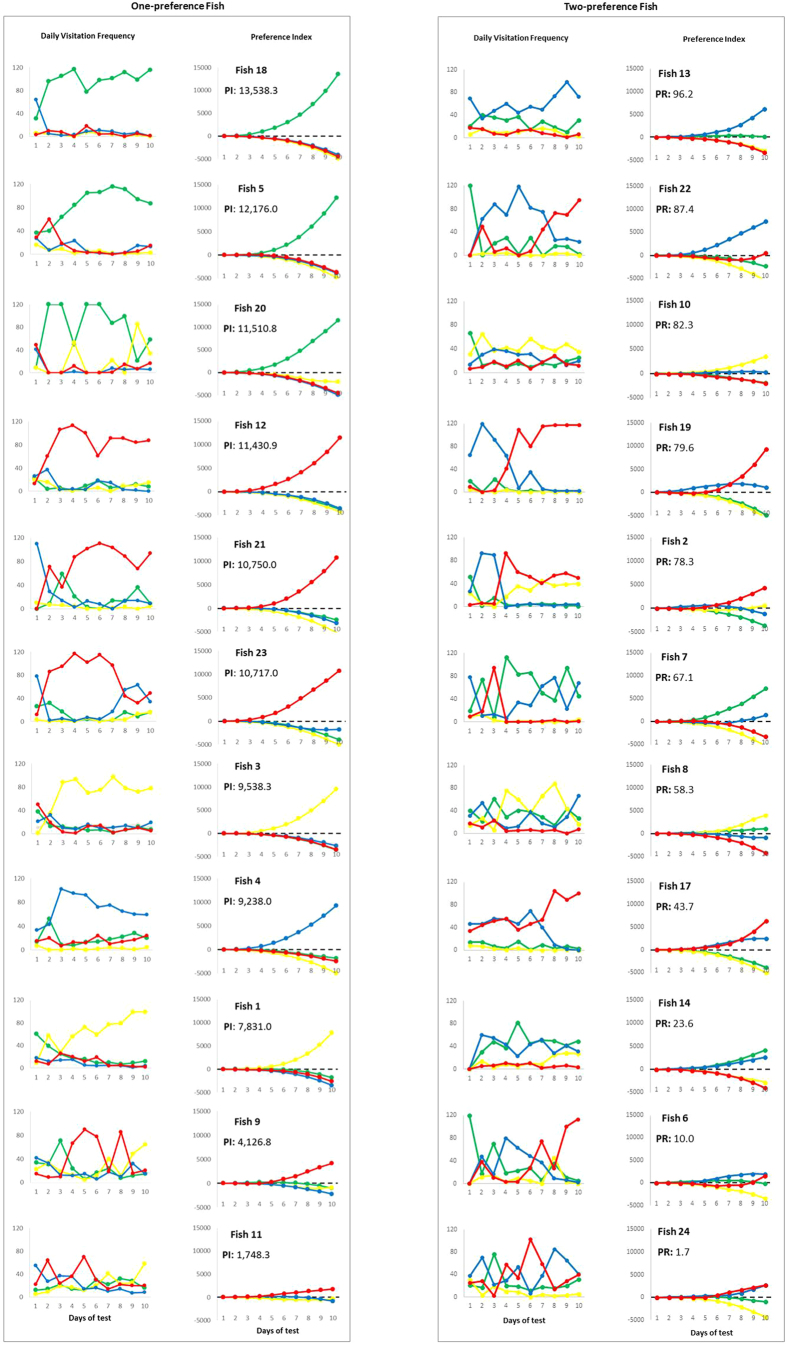
Individual profiles of preference (positive PIs) and non-preference (negative PIs) responses. PI = preference index (one-preference fish); PR = preference rate (two-preference fish). PI and PR for the last test are indicated.

The steps for calculations of choice and/or preference profiles are exemplified in Table 1, based on Fig. 2 reasoning and with data for fish 1 (Fig. 3, left panel).

The daily frequencies of visitation to each background colour (step 1) were summed per item (cumulative frequency; step 2). In step 3, we calculated the area above the cumulative-frequency line (as shown in Fig. 2). This area differentially weighs each frequency of choice in subsequent tests and increases the value of frequencies obtained in the most recent tests. Step 4 calculated the cumulative areas obtained in step 3. In step 5, we calculated an expected area (ExA); that is, we calculated the mean of the cumulative areas obtained between colours for the same fish on the same day of testing. This mean area was used as a reference to indicate how areas for each colour behave in relation to this mean area. This information was presented in step 6 (cumulative area–ExA). For each test day, we could detect the position of the cumulative area of each colour respective to a mean value expected if the area was completely random (similar to what is used in the chi-square test and also used by Larrinaga^39^, in a study of feed consumption). The preference index was assumed as the cumulative values obtained in step 6. The definition of the PI is obtained in each test (each day in this study), and we considered the final PI obtained in the last test performed. This rationale was assumed because the last test summarizes a given animal’s interaction history with the item of choice.

The preference rate (PR) was calculated for fish that exhibited more than one preference item (that is, more than one positive PI). First, we calculated the percentage of PI (each preferred colour) in relation to the sum of all positive PIs detected. For two preference items, the PR was then calculated as the lowest %PI subtracted from the highest %PI. For more than 2 preferences, the PR was calculated as the highest %PI minus [(100–% highest PI)*(Number of Preferences–1)]. This equation subtracts from the highest %PI a higher value as the fish demonstrates a greater number of preferred items.

## Results

**Figure 4 f4:**
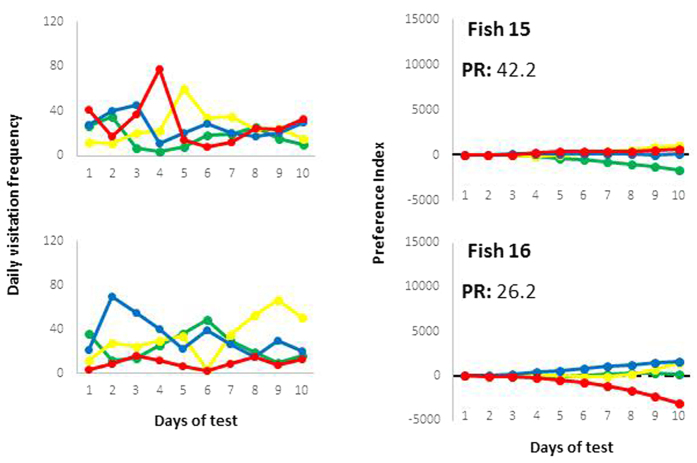
Three-preference fish. Individual profiles of preference (positive PIs) and non-preference (negative PIs) responses. PR values for the last test are indicated for each fish.

The frequencies of individual fish visitation to each compartment, their daily PI values over time and their PR values are displayed in Figs 3, [Fig f4] and 4. For all fish, we considered positive PI values as preferences and negative PI values as non-preferred items in the last choice test (10^th^ day). The one-preference fish are ranked in descending order according to their PI (Fig. 3) values in the last choice test. The more than one-preference fish are ranked in descending order according to their PR values in the last choice test, indicating 2 (Fig. 3, right panel) or 3 (Fig. 4) preference responses. For example, fish 2 (Fig. 3, right panel) exhibited a preference for the yellow colour only on the 9^th^ and 10^th^ days, but this colour was assumed as a preferred colour based on the whole 10-day evaluation period. The PI value is used to contrast interindividual profiles when fish expressed only one preference response. In fish with more than one preference, the PR value indicates the intensity of the difference between the first and other preferences in a same individual, thus contrasting interindividual profiles.

From the 4 colours for choice, 11 fish (45.8%) preferred only one colour. From fish that preferred 2 colours, 8 fish (33.3%) clearly preferred only one colour and 3 fish (12.5%) similarly preferred 2 colours. Two fish (8.3%) similarly preferred 3 colours. That is, in 19 fish (79.2%) out of 24 we could clearly identify one preferred colour. Moreover, we did not detect one colour clear-cut preference from all fish. From the 11 one-preference fish, blue was preferred once (9.1%), yellow twice (18.2%), green 3 times (27.3%) and red 5 times (45.4%). From the 11 two-preference fish, yellow was preferred 3 times (13.6%), green 4 times (18.2%), red 6 (27.3%) and blue 9 (40.1%) times. The three-preference fish preferred yellow and blue 2 times each (33.3% each), green and red once each (16.7% each).

## Discussion

To offer better conditions to confined animals, we must determine what these animals want^10^. In this issue, most studies have evaluated animal preferences considering choice and preference as synonymous terms *e.g*.^12^,^13^,^14^,^15^,^16^,^17^. However, to improve welfare, the animals should want these conditions not only momentarily, but over days because choices might differ during subsequent tests^18^,^19^. Here, we presented a history-based method to identify animal preferences from a set of successive choice tests. A PI and a PR are provided, for which data from successive choice tests can be added at any time, thus gradually providing appropriate weight to the results of successive tests for the overall inference of preference. This information allows us to separate fish by number of preferred items. Among fish that expressed preference for only one item (one-preference fish) (Fig. 3, left panel), the PI values differentiated individuals according to the intensity of the preferred item (in sequence, from fish 18 to 11 in Fig. 3, left panel). Among fish that expressed preference for two or more items (more than one-preference fish), the PR value indicates how strongly the most preferred item is relative to the other preferred items for the same fish. Therefore, PR differentiates individuals based on the intensity of differences between their strongest preference and the others. Thus, the fish in Fig. 3 (right panel) preferred fewer items than did fish in Fig. 4.

Although we used frequencies of visitation instead of time spent in each compartment, we used an instantaneous sampling method, which is sensitive to variations in time spent and visitation frequency in each available item when the interval period is short and the observed behaviour is frequent^36^. In this study, fish position was registered every 30 s during 1 h per test, providing a scanning measurement of 120 observations that could detect a reliable picture of the fish behaviour in the test apparatus. Moreover, in practical terms, inspections in captive conditions (*e.g.*, zoos) are more easily performed when quantifying frequencies than time spent in items in choice tests (assumption #5 in Methods).

Colour preference for fish has been demonstrated by several studies; however, most studies demonstrate one preference for the entire sample of fish^30^,^40^,^41^,^42^. These studies, however, used a small number of daily tests (usually 1 to 4 tests), whereas we used 10 successive days of testing. Moreover, the decision to identify a preferred item in these cited studies was based on the mean and total or cumulative frequencies/time spent; that is, data from the first day of testing have the same weight as a frequency obtained in a test performed several days later. In our method, the determination of the areas corrected this by assigning greater value to each frequency obtained in more recent tests (see Fig. 2 and fish 19 in Fig. 3, right panel). This assumption was made (assumption #2 in Methods) because recent tests reflect the most recent state of the individual and preferences can change over time. However, we did not consider only the last test because those data may contain a spurious decision by the animal. Thus, as we have incorporated the entire history of frequencies in successive tests and given more weight to the most recent tests, we implied that our results are a balance between the most consistent response of the animal over time and its momentary state in the last test, thus supposedly reflecting a more natural response. In practical terms, an animal keeper might be interested in knowing what the animal wants to adjust its holding condition. If he/she uses the PI concept described here, the result of the choice test is balanced by the whole set of previous tests, thus reflecting the actual choice, but does not ignore the previous choices of that animal. Therefore, with only one test each time (*e.g.*, once a day or a week) the animal keeper gets a better impression about the wants of that animal. Moreover, it is more reliable to include more short-term choice tests than long-term tests. This emphasizes the need for knowing individual animal wants as much as possible.

The assumption that positive PIs indicate preference items, whereas negative values are non-preferred items, is not a cabalistic one. A positive PI indicates the animal chose that item more frequently above the expected mean (ExA) relative to the other items. This information represents a relative response and a differentiated performance. Of course, this procedure will always result in animals showing at least one preferred and one non-preferred item. This assumption makes sense and is reasonable, as a preference response in a multiple choice test should always be relative to the other available items in that specific test. For example, a preference response for blue colour may supposedly appear in a choice test including blue, green, yellow and red colours but may not appear when another colour, *e.g.*, purple, is also offered as an available item and is preferred instead of blue. Moreover, the responses we identified here through negative PI values as non-preferred items may be similar to the “dispreferred” options of food colour as described by Larrinaga^39^.

Once an animal expresses a preference for only one item, the magnitude of its preference can be easily inferred from the magnitude of PI (fish in Fig. 3, left panel). However, this index cannot be contrasted with animals that chose more than one item because to choose only one item is qualitatively very different from choosing two or more items (assumption #4 in Methods). Thus, for the other animals, PR is provided to indicate a response relative to the other preferred items (Figs 3, right panel, and 4). More specifically, PR indicates how strongly the animal prefers the most preferred item compared with the other preferred items. We also assume that preference for only two items gives the animal a more clear decision-making process than when more preferred items are detected, which was also included in the PR calculations (by including number of preferences minus 1 in the calculations, as specified in the Methods).

To our knowledge, only one published study^43^ used a PI to identify the intensity of choices. This index was based on the difference of time spent in each available item. However, only two trials were examined, and preference was investigated at a group not individual level. Based on the same principle of choices, our method examines this issue more deeply by clearly separating preferences from non-preferences and by individually identifying the intensity of such responses.

The use of choice tests to infer preferences requires caution. For example, the chosen item might draw the animal’s attention–the choice is only a perceptual consequence^44^. Moreover, choice tests repeated over time (necessarily involving previous experience) and the nature of the stimulus are also intrinsic factors in preference tests that might change animal’s preference. Part of these limitations can be minimized by increasing the number of successive choice tests. The PI solved this problem in our study as variations of fish choices over time (graphs on the left in Figs 3, 4) were transformed into more consistent profiles of PI overtime (gradually increasing PI). Only one fish (Fig. 3, right panel, fish #19) exhibited inversion of the most preferred item during observations. This finding is expected (assumption #2 in Methods) and was clearly detected from PI. Note that PI detected this inversion 3 tests later in relation to the raw frequency data, a result from the high impact of the first days when fish chose strongly the blue environment. This phenomenon occurs because PI change more slowly than do direct observations of frequency of choices. Thus, PI is more robust and less sensitive to immediate changes, as we should assume when searching for preferences instead of momentary choices (non-preferences). Because of this property of PI, very confusing results are obtained with raw frequencies (*e.g.*, fish 2, 8, 10, and 24 in Fig. 3, right panel) and can be clarified using PI calculations. The limitation related to the previous experience in a serial set of tests cannot be abolished, but the history-based approach adopted here assumes that preference, except in strongly genetic preferences, is the result of different intrinsic (learning and physiological needs) and extrinsic forces (nature of the stimulus and environmental context). That is, PI does not reveal preference causes, but the preference in a history of animal experiences.

According to Kirkden & Pajor^45^, choice tests also indicate animal’s motivation to get a resource. Our individual data show that animals usually inspected all the colour items and stayed more frequently in one or few of them in each daily test. That is, higher frequencies in one item should mean more motivation for that item. The PI described here should thus indicate such a motivation, but in a history-based approach. The consumer surplus concept applied to motivational strength^46^ is an interesting approach for which the present PI could be helpful because this index somehow incorporate motivational forces. Motivation for a resource depends on the resource type, once the animal’s choices might be influenced by its satiation to the resource^46^. Thus, caution is required to compare PI values among resources.

## Additional Information

**How to cite this article**: Maia, C. M. and Volpato, G. L. A history-based method to estimate animal preference. *Sci. Rep.* **6**, 28328; doi: 10.1038/srep28328 (2016).

## Figures and Tables

**Table 1:** Preference Index calculations.

Test Number (tn)	STEP 1	STEP 2	STEP 3	STEP 4	STEP 5	STEP 6	STEP 7
	Raw Frequency (RF)^1^	Cumulative frequency^2^	Area^3^	Cumulative area^4^	Expected Area^5^ (ExA)	Variation of cumulative area from the ExA^6^	Preference Index (PI)^7^
1	9	9	4.5	4.5	12.4	−7.9	−7.9
2	57	66	85.5	90.0	55.5	34.5	26.6
3	28	94	70.0	160.0	113.6	46.4	73.0
4	56	150	196.0	356.0	208.1	147.9	220.9
5	72	222	324.0	680.0	326.3	353.8	574.6
6	59	281	324.5	1004.5	451.4	553.1	1127.8
7	77	358	500.5	1505.0	607.4	897.6	2025.4
8	79	437	592.5	2097.5	785.5	1312.0	3337.4
9	99	536	841.5	2939.0	1023.5	1915.5	5252.9
10	99	635	940.5	3879.5	1301.4	2578.1	7831.0

This procedure is carried out for each colour. Example extracted from fish 1, yellow colour.

^1^Frequencies obtained in each test.

^2^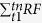.

^3^Areas calculated as shown in Fig. 2.

^4^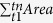.

^5^Mean of cumulative areas from each colour in the respective test number and fish. In this table, we show data only for the yellow colour; the ExA is calculated from data of all tested colours at tn for fish 1.

^6^Data from Step 4–Step 5.

^7^Cumulative data of Step 6.
